# Epidemiology and Outcomes of Late-Onset Neonatal Sepsis in Preterm Infants in a Tertiary Hospital

**DOI:** 10.3390/children12050532

**Published:** 2025-04-22

**Authors:** Katerina Kaffe, George A. Syrogiannopoulos, Efthimia Petinaki, Maria Goudesidou, Anna Kalaitzi, Antonios Gounaris, Ioanna N. Grivea

**Affiliations:** 1 Neonatal Intensive Care Unit (NICU) of the Department of Pediatrics, University of Thessaly, University General Hospital of Larissa, 413 34 Larissa, Greece; syrogian@otenet.gr (G.A.S.); mgoudesidou@uth.gr (M.G.); ankalaitzi@uth.gr (A.K.); agounaris@med.uth.gr (A.G.); iogrivea@med.uth.gr (I.N.G.); 2 Department of Microbiology, University of Thessaly, University General Hospital of Larissa, 413 34 Larissa, Greece; petinaki@uth.gr

**Keywords:** late-onset sepsis, culture-negative late-onset sepsis, preterm infant, neonatal outcome, neonatal morbidity, neonatal mortality

## Abstract

**Background/Objective:** Late-onset sepsis (LOS), a systemic infection occurring after 72 h of life, is a significant issue of morbidity and mortality in preterm neonates. Nevertheless, in this population, cultures frequently remain negative, even in the presence of typical clinical signs of sepsis. **Materials and Methods:** This single-center, retrospective study included preterm infants with a birth weight (BW) < 1500 g and/or a gestational age (GA) ≤ 32 weeks, diagnosed with culture-negative LOS (CNLOS) and culture-proven LOS (CPLOS). The study aimed to determine the incidence of these conditions, describe the frequency of isolated pathogens, and compare clinical profiles, antibiotic usage, morbidity, and mortality between these two groups as well as a no-sepsis group. **Results:** Among 277 infants, 30 (10.8%) had CPLOS, 83 (30%) had CNLOS, and 164 (59.2%) had no sepsis. Significant differences were found between the groups regarding BW, GA, hospitalization duration, morbidity, and mortality (*p* < 0.001). CNLOS and CPLOS did not differ in terms of mechanical ventilation or central line use. However, CPLOS infants had a higher rate of thrombocytopenia (*p* < 0.001), inotrope use (p = 0.006), and mortality (*p* < 0.001) compared to CNLOS infants. The duration of antibiotic treatment was similar between groups [median DOT (IQR): 20 (14–33) vs. 20 (14–35), p = 0.935]. In the CPLOS group, Gram-negative pathogens were isolated in 42.4% of infants, with Klebsiella oxytoca being the most common; Gram-positive organisms in 36.3%; and fungi in 21.2%. **Conclusions:** LOS, whether culture-proven or not, was associated with neonatal morbidity and mortality. CPLOS was linked to a worse prognosis, while CNLOS was also frequently diagnosed and associated with increased antibiotic use in Neonatal Intensive Care Units (NICUs).

## 1. Introduction

Despite the rapid progress in neonatal care in recent years, late-onset neonatal sepsis (LOS), defined as a systemic infection beyond 72 h of age, remains an important cause of morbidity and mortality, especially in very low birth weight (VLBW; weight < 1500 gr at birth) neonates and in very premature neonates (≤32 weeks of gestational age) [1,2]. Moreover, a major issue in neonates, especially premature ones, is the phenomenon of negative blood cultures in infants with clinical signs of sepsis. Remarkably, most evaluations for LOS do not isolate pathogenic microorganisms in preterm infants, and a negative culture does not entirely exclude a septic episode [3]. Consequently, LOS is classified as either CPLOS when confirmed by blood or cerebrospinal fluid (CSF) culture or CNLOS, which describes episodes with clinical and laboratory signs of infection with the absence of pathogenic microorganisms in cultures, treated with antibiotics for at least five days [4]. Recent studies among VLBW neonates estimated a current CPLOS frequency of 8.9%, compared to the value of 25% recorded in the past [5]. This incidence is significantly higher in premature infants with extremely low birth weight (<1000 gr -ELBW) [6]. On the other hand, the incidence of CNLOS ranges from 6.7 to 17.4% in preterm infants [4]. The incidence of LOS, proven with culture or not, has been inversely related to gestational age and birth weight [7]. In addition to gestational age and birth weight, other well-recognized risk factors for LOS are the use and the duration of central lines, mechanical ventilation, total parenteral nutrition, poor enteral feeding, and other comorbidities of prematurity, such as patent ductus arteriosus and necrotizing enterocolitis [8,9]. As for the main pathogens responsible for CPLOS, according to studies, they are coagulase-negative staphylococci (CoNS), *Escherichia Coli* and *Klebsiella* species (spp) [3,5]. With regard to the influence of LOS on neonatal outcomes, CPLOS has been associated with an increased risk of death, prolonged hospitalization, and long-term neurodevelopmental impairment [3,10,11]. Regarding the mortality in CPLOS, this depends on the pathogenic organism. So, mortality can be reached in up to 15%, 20%, and 31% of infants with Gram-positive, Gram-negative, and fungal CPLOS, respectively, especially in ELBW infants [3]. Similarly, CNLOS is correlated with poor neurodevelopmental and growth outcomes in early childhood, but with lower mortality incidence, less than 4% [4,11,12]. Besides the above, the negative impact of LOS on increased antibiotic use in NICUs is also significant. CNLOS is related to almost 10 times higher antibiotic use compared to CPLOS, and it is proven that higher antibiotic use in preterm infants has been associated with neonatal mortality and morbidity associated with necrotizing enterocolitis (NEC), bronchopulmonary dysplasia (BPD), severe neurologic injury, and retinopathy of prematurity (ROP) [13,14,15]. The problem of LOS and its impact on both the immediate and long-term morbidity of very preterm infants, combined with the need to record the frequency of LOS, culture-proven or not, in preterm infants in Central Greece, was what motivated us to conduct this survey.

This study aimed to investigate the incidence of CPLOS and CNLOS in a cohort of very preterm infants with BW < 1500 gr and/or GA ≤ 32 weeks in a tertiary NICU and discriminate differences of neonatal outcomes between a CPLOS group, a CNLOS group, and a No LOS group during the same period.

## 2. Materials and Methods

### 2.1. Study Design

We performed a 4-year retrospective study of all cases of CPLOS and CNLOS in a cohort of neonates with BW < 1500 gr and/or GA ≤ 32 weeks in a single-level ΙΙΙ Neonatal Intensive Care Unit (NICU). We recorded all premature infants with the aforementioned characteristics who were admitted to the NICU of the University of Thessaly between 1 January 2014 and 31 December 2017. Data collection was performed from the medical files while maintaining the anonymity of the infants during the analysis. All authors had access to information during and after data collection. Our NICU, the reference NICU of central Greece, has a capacity of 20 intensive care beds and 10 additional intermediate care beds. Intermediate care beds are for neonates who do not require mechanical ventilation or continuous positive airway pressure support and have no need for inotropic drugs to sustain blood pressure. Our infection control practice includes hand hygiene, mask and gown use, immediate infection management, active surveillance of healthcare-associated infections (HAI), particularly central-line-associated bloodstream infections (CLABSIs) and the implementation of bundles for the prevention of CLABSIs, but in the early stages. The infection control measures that were implemented were the same throughout the four years of the study, and there was no antibiotic surveillance program in place during the study. Additionally, the approach to diagnosing and managing infections, as well as the antibiotic regimen used, remained the same during the study.

The primary aim of our investigation was to determine the incidence of CPLOS and CNLOS in this group of neonates. We also recorded the clinical characteristics of infants who did not suffer from LOS, those who had CNLOS, and those with CPLOS, to investigate any differences in the clinical profiles among these groups. Furthermore, we compared these three groups in terms of mortality and morbidity, focusing on complications associated with systemic inflammation, such as periventricular leukomalacia (PVL), severe retinopathy of prematurity (ROP), and bronchopulmonary dysplasia (BPD). Then, a comparison of the characteristics of CNLOS and CPLOS episodes was conducted based on the use of antibiotics, the duration of mechanical ventilation, and the duration of central lines, and their severity, as interpreted by mortality, the presence of thrombocytopenia, the use of inotropes, and the need for transfusions. Finally, in the CPLOS group, we determined the frequency and the characteristics of the isolated pathogens.

### 2.2. Data Collection

A standardized database was initially created, and accurate demographic, clinical, and laboratory data of preterm infants with BW < 1500 gr and/or GA ≤ 32 weeks were recorded retrospectively. Τhen, the infants were divided into three groups depending on whether they developed late-onset sepsis during their hospitalization or not. Group A included infants without LOS; group B infants with CNLOS; and group C infants with CPLOS. All data collection followed the accepted definitions.

### 2.3. Definitions

CPLOS was defined as sepsis with a positive result on one or more blood or cerebrospinal fluid (CSF) cultures obtained after 72 h of life. Positive blood culture results were defined as the isolation of a Gram-positive, Gram-negative, or fungal organism. Cultures which were positive for possible contaminants, mainly coagulase-negative staphylococci, were considered indicative of infection if the neonates fulfilled all 3 of the following criteria: (1) 1 or more infection-related clinical manifestations (apnea, increased oxygen demand, malnutrition/abdominal distension, CRT > 2 s); (2) 2 positive blood cultures drawn within 2 days of each other, or 1 positive blood culture combined with an abnormal white blood cell count or CRP level; (3) effective antibiotics received for ≥5 days [16]. The date of CPLOS onset was defined as the date of positive blood culture. An antibiotic course consisted of 1 or more uninterrupted antibiotic days.

CNLOS was diagnosed when all of the following criteria were fulfilled: (1) two or more infection-related clinical manifestations (apnea, increased oxygen demand, malnutrition/abdominal distension, CRT > 2 s); (2) an increase in CRP > 1 mg/dL, or WBC < 5000/μL or >25,000/μL; (3) antibiotics used or intended for ≥5 days; (4) negative blood culture with no or negative cerebrospinal fluid culture; (5) no evidence of concurrent focal infection, including pneumonia, urinary tract infection, and necrotizing enterocolitis [4]. Central-line-associated bloodstream infection (CLABSI) was defined as any bacteremia on a single blood culture for organisms not commonly present on the skin and two or more blood cultures for organisms commonly present on the skin in a neonate hospitalized for at least two days and a central catheter in place at the time of infection or within two calendar days before, with the infection not be ascribable as secondary to other infections [17]. A significant challenge is differentiating CNLOS from non-infectious inflammatory syndromes. In preterm infants, distinguishing between infections and non-infectious inflammatory syndromes is particularly difficult due to their immature immune systems and overlapping clinical presentations [18]. However, we used a combination of clinical symptoms, such as poor feeding, lethargy, respiratory distress, tachycardia, hypotension, or jaundice, along with laboratory markers, primarily a rapid rise in CRP and, most importantly, a rapid response to antibiotic therapy, to differentiate the two conditions.

The CRIB II score, which evaluates the severity of illness and the risk for mortality, was calculated as the sum of the following three parameters: sex, BW (gr), and GA (weeks) (first parameter); the maximum base excess (second parameter); and temperature upon admission (°C) (third parameter) [19]. This index is a useful and easy tool for the prediction of mortality and short-term morbidity related to prematurity in neonates with GA ≤ 32 weeks [20]. The parenteral antibiotics administered to the neonates during hospitalization were recorded. Every calendar day a patient received 1 antibiotic equaled 1 DOT (Day οf Therapy) [21]. Also, the ASI (antibiotic spectrum index) for each antibiotic course was calculated using the Gerber et al. scoring system and summed for each patient’s NICU stay; briefly, the ASI assigns a point for activity against 13 clinically relevant pathogens with an additional point for activity against multidrug-resistant organisms [22,23]. First, the ASI/AD-1 values were calculated for each neonate of the three groups based on antibiotics received per antibiotic day for suspected early-onset sepsis (EOS). Suspected EOS was defined as the presence of all of the following conditions: maternal risk factors for sepsis (preterm onset of labor, prolonged rupture of membranes ≥18 h, group B streptococcus (GBS) colonization in the current pregnancy, GBS bacteriuria, invasive GBS infection in a previous infant, temperature ≥ 38 °C, and clinical signs of chorioamnionitis); perinatal risk factors (fetal tachycardia); compatible clinical symptoms for neonatal sepsis in the first 3 days (temperature instability, apnea, tachycardia, and/or tachypnea) laboratory findings (WBC < 5000/μL and/or CRP > 1 mg/dL); and antibiotics administered for ≥7 days [24,25]. The ASI/AD-2 values were calculated for each neonate of the groups of CNLO and CPLOS during the LOS episodes as previously described.

Regarding the morbidity of prematurity, we defined BPD as oxygen dependency at 36 weeks postmenstrual age, PVL as the presence of periventricular cysts on cranial ultrasound or cranial ΜRI, or increased periventricular echogenicity persisting for more than seven days according to the classification by DeVries, as well as severe ROP, ROP stage ≥ΙΙΙ, according to the International Classification of ROP. The classification of intraventricular hemorrhage (IVH) was based on the Papille IVH classification [26]. NEC was staged according to the modified Bell criteria [27]. Apnea was defined as cessation of breathing for 20 s or longer or a shorter pause accompanied by bradycardia (<100 beats/min), cyanosis, or pallor [28]. Finally, extrauterine growth restriction (EUGR) was defined as weight < 10th percentile or Z score < −1.28 either on discharge or at 36–40 wk postmenstrual age [29,30].

### 2.4. Statistical Analysis

Our study was designed as a retrospective categorical study with three variables. The data were summarized by using descriptive statistics. Continuous variables are presented as the mean (standard deviation) for normally distributed data and the median (interquartile range—IQR) for non-normally distributed data. The assumption of normality was investigated by using a histogram for each variable. Statistical significance was defined as *p* < 0.05. Categorical variables are presented as absolute and relative values and were compared with Pearson’s chi-square test. Continuous variables, which followed a normal distribution, were tested by ANOVA. For multiple comparison adjustment, a *p*-value of 0.017 was considered significant according to the Bonferroni method for multiple comparisons. Continuous variables, which followed a non-normal distribution were tested with a non-parametric test. All statistical tests were two-sided. Statistical analyses were performed using ΙΒΜ SPSS statistics version 29.0.0.0 (241).

## 3. Results

We retrospectively studied 295 infants with BW < 1500 gr and/or GA ≤ 32 weeks (144 males) who were hospitalized during 2014–2017 (four years). The mean GA was 29.7 ± 2.7 weeks, and the mean BW was 1299 ± 350 gr.

**Figure 1:**
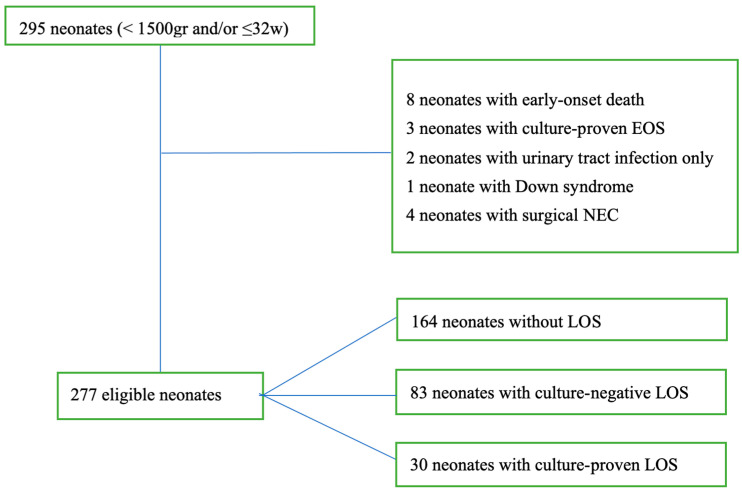
Patient populations in the present study.

Most neonates were born at the University Hospital of Thessaly (78.8%) by cesarian section (72%). In 74.9% of pregnancies, antenatal steroids were administered. The causes of preterm delivery were infection (42.8%), bleeding (11%), intrauterine growth restriction (14.8%), preeclampsia (6.4%), intrauterine growth restriction complicated by preeclampsia (2.8%), severe fetal malformations (3.9%), multiple pregnancies (10.6%), and other diseases in the mother (2.8%), while in 4.9% of cases, the cause of prematurity was infection with one or more of the above risk factors. Total mortality (early and late) was estimated to be 8.1% (24/295) with variation per year from 3.9 to 16%. In the total cohort, the estimated frequencies of the three major morbidities of prematurity, BPD, PVL, and ROP, were 30.8%, 22.4%, and 10.5%, respectively. Eighteen neonates with early onset death and proven early-onset sepsis, urinary tract infection, genetic syndromes, and necrotic enterocolitis (NEC) with perforation were excluded from the analysis (Figure 1). Of the 277 eligible neonates, 164 (59.2%) did not have any episodes of late-onset sepsis (No LOS), 83 had CNLOS, and 30 had CPLOS, resulting in an estimated incidence of CNLOS and CPLOS of 30% and 10.8%, respectively (Figure 1). The comparative maternal and peripartum variables and the neonatal clinical characteristics and outcomes are depicted in Table 1 and Table 2.

Statistical comparison of the three groups showed significant differences in BW and GA. Regarding the maternal and peripartum variables concerned, there was no difference in the type and the place of delivery, antenatal steroid administration, the frequency of diabetes mellitus (DM), and intrauterine growth restriction (IUGR). However, there was a statistically significant difference in the presence of maternal infection and the need for resuscitation at birth. The comparison of neonatal characteristics revealed significant differences in the CRIB II index, days of full enteral feeding, duration of total parenteral nutrition, the presence of central lines—whether umbilical or PICC—and the duration of mechanical ventilation and oxygen administration. In addition, there was a significant difference in common problems of prematurity such as respiratory distress syndrome, patent ductus arteriosus, apnea of prematurity, IVH, and the need for transfusion with packed red blood cells (PRBCs) due to anemia of prematurity. No difference in jaundice emerged between groups, but there was a difference in the duration of phototherapy, which was significantly higher in the CPLOS group (*p* < 0.001). The three groups differed in the frequency of probable early sepsis. However, the assessment of these groups by the DOT and ASI/AD indices did not reveal differences in antibiotic administration. As for major morbidities, mortality, and the duration of hospitalization, the analysis revealed differences between groups, apart from EUGR frequency.

Comparing the two LOS groups, one by one, the CPLOS and CNLOS groups had similar characteristics regarding antibiotic use and the duration of mechanical ventilation and central lines. However, inotrope administration, the number of required PRBC transfusions, the occurrence of thrombocytopenia, and mortality during hospitalization significantly differed (Table 3). In the case of thrombocytopenia, a difference in severity was shown. The mean lowest platelet count in thrombopenic infants in CNLOS was 80,500 ± 26,506/μL, significantly higher compared to the CPLOS group, which had a lowest mean platelet count of 25,666 ± 18,739/μL (*p* < 0.001).

The distribution of isolated pathogens in positive blood or CSF cultures in LOS episodes is detailed in Table 4. The number of isolated microorganisms in these 30 neonates was 33. In a CLABSI episode, two Gram-negative pathogens were recovered from the same culture and two infants presented with two separate episodes of confirmed LOS. Of these 32 episodes of LOS, 14 were CLABSIs. The most frequently isolated pathogens were Gram-negative ones (14/33, 42.4%), with *Klebsiella oxytoca* being the most prevalent. Gram-positive organisms closely followed (12/33, 36.3%), with the main representative being coagulase-negative staphylococci (Cons). The frequency of fungi was calculated to be 21.2% (7/33), with *Candida* spp. being the most prevalent. All infants with invasive fungal infection were ELBW and extremely premature, with mean BW and GA of 653 ± 147.6 gr and 25.1 ± 1.2 wk, respectively.

## 4. Discussion

LOS remains a major issue in the NICU among all birth weight groups and a threat to both survival and long-term neurodevelopmental outcomes, especially in very preterm neonates [31]. Our study confirmed the impact of well-recognized risk factors for LOS, such as lower gestational age and birth weight, the placement of central catheters, the duration of total parenteral nutrition (TPN), and mechanical ventilation. These results are consistent with those of previous studies. It is well proven that the incidence of LOS is inversely proportional to GA and BW [32]. Moreover, the duration of TPN is an independent risk factor for LOS irrespective of the causative pathogen (OR 1.1; 95% CI 1.04–1.216; *p* = 0.003) [9]. Similarly, central lines and their dwell time during the first two weeks of life are important risk factors for LOS [3,32].

Regarding maternal and perinatal history, our analysis revealed that only two parameters, maternal infection and the need for resuscitation at birth, were significant risk factors for LOS. These findings are in concordance with a recent metanalysis by Beck et al. which revealed chorioamnionitis as a risk factor for LOS among neonates: 22% in the presence of histologic disease, and 26% among clinical chorioamnionitis-exposed neonates, particularly in the case of maternal infection which is due to Gram-negative bacteria [33].

The present study also unveiled statistically significant differences among these groups for all major complications of prematurity, except for EUGR. These differences have a pathophysiological basis, as PVL, BPD, and severe ROP are three neonatal morbidities that are directly associated with inflammation due to LOS, whether confirmed by culture or not [4]. The previous literature also supports our findings. PVL, which directly affects the neurodevelopmental outcomes in very preterm neonates, has been described to be much more likely both in CPLOS and CNLOS [34,35]. Furthermore, Huncikova et al., showed a high correlation of severe BPD with both culture-proven (aOR1.7; 95% CI 1.3 to 2.2) and culture-negative (aOR1.6; 95% CI 1.3 to 2.6) LOS, while only CPLOS was associated with increased odds of cystic PVL and severe ROP (aOR:2.2; 95% CI 1.4 to 3.4 and 1.8; 95% CI 1.2 to 2.8, respectively) in very preterm infants [36]. In our study, EUGR ranged from 29 to 54%, a rate which is similar to those of previous studies on European populations: EUGR among very preterm neonates was in the range of 24–60% when using the Fenton percentiles and 39–72% when using Fenton delta Z-score [37]. The lack of significance of EUGR for LOS in our cohort could be attributed to the ‘aggressive’ feeding protocols, particularly in the CNLOS and CPLOS groups implemented in our NICU [38].

The incidence of CPLOS in our report is estimated to be 10.8%, a result which confirms the improvement in CPLOS rates during the last decade, according to recent studies. While one decade ago, CPLOS incidence ranged between 21 and 27% [10], more recent studies have recorded even lower rates of CPLOS. Vestraete et al. and Hornik et al. reported LOS incidences for VLBW neonates of 15.3% and 12%, respectively [31,39]. More recently, Flannery et al. reported that 8.9% of very preterm neonates with BW < 1500 gr and/or GA < 29 wk had confirmed late-onset sepsis [5]. Additionally, Mukhopadhyay et al. reported that LOS prevalence ranged from 8 to 37% among ELBW and very preterm neonates [34]. Finally, in a multicenter study of 21 NICUs in Norway, Huncikova et al. found an incidence of 9.3% in CPLOS [36].

CNLOS incidence is 30%, which is higher than that of confirmed LOS. ‘CNLOS predominance’ is consistent with results from other studies that compared preterm groups with CNLOS and CPLOS; in those studies, the diagnosis of CNLOS was approximately twice to three times more frequent than that of CPLOS [4]. Siyan Jiang et al. recorded an incidence of culture-negative sepsis of 6.7% and 17.4% in preterm infants under 34 wk and ELBW neonates, respectively, while Mukhopadhyay et al. found a higher incidence of 23–63% in the group of ELBW neonates [4,34]. On the other hand, Huncikova et al. recorded a lower incidence of CNLOS of 5.3% in preterm infants < 32 wk and 11.1% in extremely preterm infants (<28 wk) [36].

The higher value of CLNOS in our report is not surprising taking into the account the definition of CNLOS; this definition, particularly when preterm neonates are included, is a combination of clinical signs, abnormal laboratory findings, and the duration of antibiotic treatment, which can lead to an overestimation of CNLOS incidence [4]. Furthermore, the low yield of blood cultures in premature neonates could be a second parameter. Hornik et al. reported that in a cohort of VLBW neonates, only 8.9% of blood cultures obtained to confirm suspected LOS were positive [39]. This finding can be explained by low-level bacteremia, insufficient blood culture, or previous antibiotic administration [3]. It is well understood by neonatologists that only one in every five evaluations for sepsis by blood culture yields a pathogen [3]. Therefore, at least one sample with 1 mL of blood should be collected for culture if infection of the bloodstream is suspected. Under these conditions, its sensitivity for the detection of bacteremia is expected to approximate 90% [40]. In addition, ‘sepsis-mimicking’ conditions such as intubation or enteral feeding intolerance, characterized by clinical instability, lead to the overdiagnosis of sepsis in preterm infants and CNLOS overestimation [41]. Finally, certain CNLOS episodes could be caused by viruses, which are increasingly recognized as causative agents of infections in NICUs. Respiratory Syncytial Virus (RSV), influenza, and parainfluenza viruses and rhinovirus may present with the clinical picture of respiratory illness or apnea, especially RSV. Rotavirus, adenovirus, and norovirus may appear with gastrointestinal illness or NEC. Rarely, viruses such as parechovirus may present with sepsis-like syndrome that is indistinguishable from CNLOS, unless the clinician is cautious with their characteristic rash. Usually, all these infections have an epidemic character in the NICU [32]. Special emphasis should be placed on cytomegalovirus (CMV), which can also present as sepsis-like syndrome, particularly in preterm neonates who are fed with fresh non-pasteurized breast milk [42,43]. The issue of viruses as causative pathogens may explain the higher incidence of CNLOS in our study, since during this study, testing for viruses was only conducted in cases with an epidemic character and for CMV in preterm infants < 32 weeks, who were fed with breast milk.

Comparing the clinical characteristics of the CNLOS and CPLOS subgroups, no difference in the duration of mechanical ventilation and central lines was found. However, there were differences among these groups in favor of CNLOS in parameters such as the use of inotropes, thrombopenia, the total number of transfusions with PRBC, and mortality. These findings reinforce literature observations describing CNLOS as a less severe “septic condition” than CPLOS [3].

In our study, the use and duration of appropriate antibiotics, as described by the DOT 2 and ASI/AD 2 indicators, did not differ significantly between the CPLOS and CNLOS groups, a finding that emphasizes the issue of antibiotic overuse in CNLOS described in other studies. The median duration of antibiotic treatment for each episode of culture-negative LOS was calculated to be 11 days while the suggested duration of the antibiotic course was 5 days [4,13]. CNLOS and antibiotic overuse have become a subject of particular interest in the recent literature, as increased antibiotic use has been associated with neonatal mortality and morbidities such as necrotizing enterocolitis, BPD, severe neurologic injury, ROP in infants without culture-proven sepsis, and NEC [44]. Another finding of our study highlights the issue of antibiotic overuse in NICUs: prior antibiotic use for possible early-onset neonatal sepsis was not statistically significant among the three groups. This further reinforces the common clinical impression that nearly all VLBW neonates receive antibiotics after birth because of the risk of early-onset sepsis [45,46].

Concerning the microorganisms isolated in our study, Gram-negative pathogens predominated slightly over Gram-positive bacteria (42.4% vs. 36.3%). This is consistent with another Greek multicenter study in which 14 NICUs participated, where the primary pathogens were Enterobacteriaceae (36%) [47]. It is traditionally known that Gram-negative pathogens are the predominant cause of LOS in developing countries [48]. According to cohorts in the United States and Europe, the most common Gram-negative pathogen was *Escherichia coli* (range 3–13%), followed by *Klebsiella* spp. (4–5%), *Pseudomonas* spp. (2–5%), *Enterobacter* spp. (2.5–21%), *Serratia marcescens* (0.8–2%), and *Acinetobacter baumannii* (0.1–2%) [3]. But it is worth noting that *Klebsiella* spp. have shown an increase according to recent reports, occurring in almost 31% of CPLOS cases [49]. Regarding mortality in CPLOS caused by Gram-negative pathogens in very preterm infants, *Pseudomonas* spp. seems to be the leading pathogen [48]. On the other hand, Gram-positive bacteria are the most common pathogens isolated in high-income countries, and more than 50% of Gram-positive bacteremia among preterm neonates is caused by coagulase-negative staphylococci (CoNS) [5,50,51,52]. Fungal organisms were isolated in about 3–10% of cases of LOS, with Candida species detected most frequently. In our study, the high incidence of fungal infections during the study period led to the initiation of the systemic prophylactic administration of fluconazole in ELBW neonates.

The basic limitation of our study, in addition to its retrospective design, was the relatively small sample from a single center. The retrospective nature of the study carries the risk of inaccurate retrieval of information, but taking into account that almost all of the authors work in this unit, this risk was significantly reduced. Our small sample size may affect the statistical power and the generalization of the results. Additionally, since the study was conducted in a single center, the findings may be influenced by specific characteristics of the population of premature infants in Central Greece or our local clinical practices. However, the single-center sampling could be a strength, since the same management was implemented for all preterm infants. Moreover, our results are similar to other recent reports of multicenter studies [4,5,34,36].

## 5. Conclusions

It is hard to answer the question of whether the diagnosis of late-onset sepsis requires a positive blood culture, especially in VLBW neonates and/or those at ≤ 32 weeks of gestational age. Our study confirmed that CNLOS was frequently diagnosed and treated with a long duration of antibiotics and was associated with the same adverse short-term and long-term outcomes as CPLOS. However, regarding mortality, CNLOS was a milder type of sepsis compared to CPLOS. This fact, in a way, alleviates and justifies, to some extent, the use of antibiotics in this “very sensitive” group of infants, since neonatologists are often called upon to treat LOS episodes quickly and appropriately due to the “threat” of death. These findings highlight the need to better define CNLOS episodes by implementing appropriate quick biomarkers of sepsis, as well as to apply more strict antibiotic stewardship measures for CNLOS in our NICUs, to shorten the inappropriate duration of antibiotic administration without defying the rules of safety. In the case of “culture-negative neonatal infections,” antibiotic treatment should be reserved for symptomatic infants with positive biomarkers or those with a clear source of infection. Our findings should be verified by larger multicenter studies using carefully designed, strict management protocols.

## Figures and Tables

**Table 1:** Maternal and peripartum variables of infants without LOS (No LOS), with CNLOS, and with CPLOS.

Characteristics (n 277)	No LOS (n 164)	CNLOS (n 83)	CPLOS (n 30)	*p*-Value
Prenatal steroids, n (%)	126 (76.8)	64 (77.1)	22 (73.3)	0.921
Cesarean section, n (%)	125 (76.2)	65 (78.3)	19 (63.3)	0.247
In-hospital delivery, n (%)	126 (76.8)	72 (86.7)	26 (86.7)	0.12
DM, n (%)	20 (12.2)	7 (8.4)	2 (6.7)	0.25
Infection, n (%)	68 (41.5)	40 (48.2)	21 (70)	0.006
IUGR, n (%)	31 (18.9)	19 (22.9)	2 (6.7)	0.139
Resuscitation at birth, n (%)	17 (10.4)	16 (19.3)	13 (43.3)	<0.001

Abbreviations: CPLOS: culture-proven late-onset sepsis; CNLOS: culture-negative late-onset sepsis; DM: diabetes mellitus; IUGR: intrauterine growth restriction.

**Table 2:** Characteristics of infants without LOS (No LOS), with CNLOS, and with CPLOS.

	No LOS (n 164)	CNLOS (n 83)	CPLOS (n 30)	*p*-Value
Male n (%)	73 (44.5)	47 (54.2)	14 (46.7)	0.351
BW (gr), mean (SD)	1508.3 (237.5)	1105.24 (269.65)	916.66 (312.65)	<0.001
BW < 1500 gr, n (%)	85 (51.8)	81 (97.5)	28 (93.3)	
BW < 1000 gr, n (%)	4 (2.4)	35 (42.2)	22 (73.3)	
GA (wks), mean (SD)	31.24 (1.5)	28.5 (2.52)	26.93 (2.43)	
GA < 29 wks, n (%)	9 (5.5)	47 (56.6)	23 (76.7)	<0.001
Neonatal course				
CRIB ΙΙ median (ΙQR)	3 (2–4)	8 (4–10)	10 (8.75–13.25)	<0.001
ΤPΝ, n (%)	155 (94.5)	83 (100)	30 (100)	0.041
TPN duration in days, median (ΙQR)	6 (5–8)	12 (10–18)	19 (12.75–28)	<0.001
Full enteral feeding in days, median (ΙQR)	9 (8–10)	14 (10–18.5)	21 (15–29)	<0.001
Umbilical catheter, n (%)	74 (45.1)	68 (81.9)	29 (96.6)	<0.001
PICC, n (%)	4 (2.4)	32 (38.6)	11 (36.7)	<0.001
Possible EOS, n (%)	47 (28.65)	53 (63.9)	27 (90)	<0.001
DOT1, mean (SD)	14.15 (3.35)	14.89 (3.31)	14.4 (3.64)	0.182
ASI/AD-1, mean (SD)	6.5 (1.04)	6.25 (1.1)	5.87 (5.5)	0.045
O2 therapy duration in days, median (ΙQR)	4 (2–9)	48 (14–73)	60 (13.75–114)	<0.001
MV, n (%)	56 (34.14)	54 (65.1)	27 (90)	<0.001
MV duration in days, median (ΙQR)	1 (1–3)	5 (2–14)	6 (3–35)	<0.001
RDS, n (%)	108 (65.9)	80 (96.4)	30 (100)	<0.001
PDA, n (%)	7 (4.3)	17 (20.5)	11 (36.7)	<0.001
Jaundice n (%)	156 (95.1)	81 (97.6)	30 (100)	0.329
Duration of phototherapy in days, mean (SD)	2.4 (1.28)	3.52 (1.77)	4.4 (1.9)	<0.001
Apnea of prematurity, n (%)	41 (25)	65 (78.3)	25 (83.3)	<0.001
Need for PRBC transfusions, n (%)	86 (52.4)	82 (98.8)	30 (100)	<0.001
Neonatal outcome				
IVH (I-IV), n (%)	25 (15.2)	24 (28.9)	13 (43.3)	<0.001
PVL, n (%)	18 (11)	31 (37.35)	15 (50)	<0.001
BPD, n (%)	18 (11)	51 (61.4)	19 (63.3)	<0.001
ROP (≥ III), n (%)	5 (3.1)	15 (18.1)	10 (40)	<0.001
ΕUGR, n (%)	46 (28.5)	25 (31.6)	11 (52.38)	0.197
Hospitalization in days, median (ΙQR)	28 (23–39)	63 (43–85)	69.5 (33–121.3)	<0.001
Mortality, n (%)	3 (1.8)	4 (4.8)	9 (30)	<0.001

Abbreviations: CPLOS: culture-proven late-onset sepsis; CNLOS: culture-negative late-onset sepsis; BW: birth weight; GA: gestational age; wks: weeks; CRIB II: clinical risk index for babies II; TPN: total parenteral nutrition; PICC: peripherally inserted central catheter; MV: mechanical ventilation; EOS: early-onset sepsis; DOT1: days of therapy for possible EOS; ASI/AD: antibiotic spectrum index/antibiotic day-number; RDS: respiratory distress syndrome; PRBC: packed red blood cells; IVH: intraventricular hemorrhage; PVL: periventricular leukomalacia; BPD: bronchopulmonary dysplasia; ROP: retinopathy of prematurity ≥ III; EUGR: extrauterine growth restriction.

**Table 3:** Characteristics of CNLOS and CPLOS episodes.

	CNLOS (n 83)	CPLOS (n 30)	*p*-Value
Number of episodes, mean (SD)	1.57 (1.71)	1.068 (0.25)	0.039
DOT2, mean (SD)	20(14–33)	20 (14–35)	0.935
ASI/AD-2, mean (SD)	10.53 (2.57)	11.04 (3.52)	0.097
Umblical catheter duration (d), mean (SD)	11.96 (4.45)	14.17 (7.33)	0.083
PICC duration (d), median (ΙQR)	14 (9–16.5)	14 (8–33)	0.461
MV duration (d), median (ΙQR)	5 (2–14)	6 (3–35)	0.435
Number of PRBC transfusions, median (ΙQR)	3 (2–7)	6 (4–12)	0.009
Thrombopenia, n (%)	8 (9.6)	12 (40)	<0.001
Use of inotropes, n (%)	28 (33.7)	21(70)	0.006
Mortality, n (%)	4 (4.8)	9 (30)	<0.001

Abbreviations: CPLOS: culture-proven late-onset sepsis; CNLOS; culture-negative late-onset sepsis; DOT2: number of days of therapy for CNLOS or CPLOS; ASI/AD-2: antibiotic spectrum index/antibiotic day; PICC: peripherally inserted central catheter; PRBC: packed red blood cells.

**Table 4:** Frequency of sepsis microorganisms.

Microorganism	n	%	Blood	CSF
Gram-positive	12	36.4	12	0
Coagulase-negative *staphylococci*	12	36.4	12	0
*Staphylococcus epidermidis*	9	27.3		
*Staphylococcus haemoliticus*	1	3.0		
*Staphylococcus warneri*	2	6.1		
Gram-negative	14	42.4		
* Klebsiella oxytoca *	3	9.1	3	0
* Klebsiella pneumoniae *	2	6.1	1	1
* Escherichia coli *	2	6.1	2	0
* Stenotrophomonas maltophillia *	2	6.1	2	0
* Pseudomonas aeruginosa *	2	6.1	2	0
* Enterobacter aerogenes *	1	3.0	1	0
* Serratia marcescens *	1	3.0	1	0
Fungi	7	21.2		
* Candida* spp.	4	12.1	4	0
* Candida albicans *	2	6.1	2	0
* Candida parapsilosis *	1	3.0	1	0

Abbreviation: CSF: cerebrospinal fluid.

## Data Availability

The data presented in this study are available on reasonable request from the corresponding author. The data are not publicly available due to privacy.
